# Spatially Resolved Kinetic Model of Parahydrogen Induced Polarisation (PHIP) in a Microfluidic Chip

**DOI:** 10.1002/cphc.202100135

**Published:** 2021-08-31

**Authors:** Sylwia J Ostrowska, Aabidah Rana, Marcel Utz

**Affiliations:** ^1^ School of Chemistry University of Southampton Southampton UK

**Keywords:** finite element modelling, homogeneous catalysis, kinetics, microfluidics, NMR spectroscopy

## Abstract

We report a spatially resolved kinetic finite element model of parahydrogen‐induced polarisation (PHIP) in a microfluidic chip that was calibrated using on‐chip and off‐chip NMR data. NMR spectroscopy has great potential as a read‐out technique for lab‐on‐a‐chip (LoC) devices, but is often limited by sensitivity. By integrating PHIP with a LoC device, a continuous stream of hyperpolarised material can be produced, and mass sensitivities of $\mathrm{pmol}\sqrt{s}$ have been achieved. However, the yield and polarisation levels have so far been quite low, and can still be optimised. To facilitate this, a kinetic model of the reaction has been developed, and its rate constants have been calibrated using macroscopic kinetic measurements. The kinetic model was then coupled with a finite element model of the microfluidic chip. The model predicts the concentration of species involved in the reaction as a function of flow rate and position in the device. The results are in quantitative agreement with published experimental data.

## Graphical abstract

We report a spatially resolved kinetic finite element model of parahydrogen‐induced polarisation in a microfluidic chip that was calibrated using on‐chip and off‐chip NMR data. The model predicts the concentration of species involved in the reaction as a function of flow rate and position in the device.

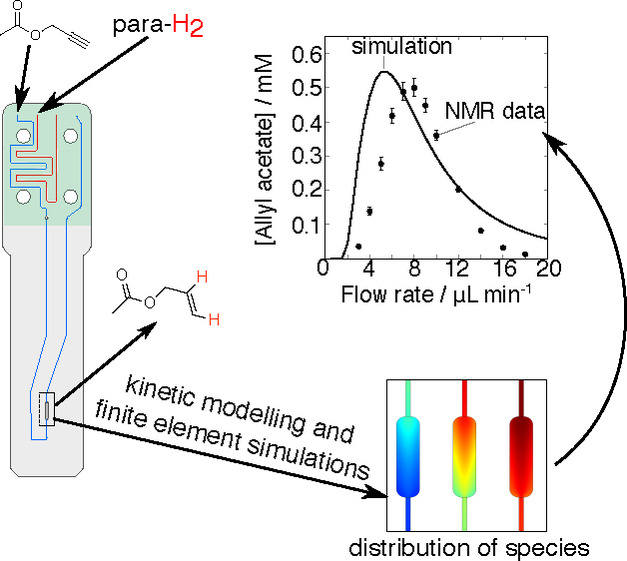

## 1 Introduction

Microfluidic lab‐on‐a‐chip (LoC) devices are increasingly used to study chemical and biochemical reactions because they allow to precisely control the reaction environment.[^1^, ^2^, ^3^] Integration of such devices with nuclear magnetic resonance (NMR) spectroscopy has several significant advantages. NMR can identify species by their distinct chemical shift patterns without the need of molecular tagging. Also, it enables non‐invasive *in operando* detection and does not disturb the thermodynamics of the system. NMR spectroscopy is based on population excess, which is only of the order of 10^−5^ in thermal equilibrium even with the highest magnetic fields available. Hyperpolarisation methods can lead to spin alignment of order unity under special circumstances. Parahydrogen ($p-\;H_{2}$ ), i. e., the singlet spin isomer of molecular hydrogen, can be used as a source of spin order.[^4^, ^5^] Parahydrogen can easily be prepared by exposing hydrogen gas to a ferromagnetic catalyst at low temperature. After heating to room temperature, the overpopulated singlet spin isomer persists for extended periods of time if contact with magnetic surfaces is avoided. The spin order can be transferred to molecules of interest through catalytic hydrogenation of an unsaturated precursor molecule. A non‐hydrogenative variant of PHIP called signal amplification by reversible exchange (SABRE) is widely used however, in this work we have have focused on the hydrogenative PHIP. Several metabolites have been prepared in a hyperpolarised state based on this principle, including lactate,^[6]^ pyruvate[^7^, ^8^, ^9^] and fumarate.[^10^, ^11^] Since hyperpolarisation is a non‐equilibrium state, it is subject to decay by spin‐lattice relaxation. Lifetimes are in the order of seconds (${}^{1}H$ polarisation) or minutes (${}^{13}C$ polarisation). After this time, a new sample needs to be prepared. Performing reactions under continuous flow allows to circumvent this constraint. Once a dynamic equilibrium is established, a constant stream of hyperpolarised material is delivered. This principle has been implemented^[12]^ at the macroscopic scale (5 mm NMR tubes) by using a hollow fibre membrane system first developed for ${}^{129}\mathrm{Xe}$ hyperpolarisation.^[13]^ Lehmkuhl *et al*.^[14]^ have proposed a 3D‐printed reactor with a serpentine pathway covered by a thin film composite membrane to facilitate hydrogen diffusion. Microfluidic flow reactors provide a cost effective approach to continuous flow PHIP by integrating catalytic hydrogenation and product detection onto a single platform at the *μ*L scale.[^15^, ^16^] Eills *et al*.^[16]^ have combined a microfluidic PHIP device with a highly optimised transmission line probe,^[17]^ and achieved signal enhancements of 1800, leading to $\mathrm{pmol}\;\sqrt{s}$ mass sensitivity. However, the yield of hyperpolarised material was low at about 2.5 %, corresponding to a concentration of 0.5 mM of hydrogenated substrate. Since further transformations and purification steps are needed after hydrogenation in order to produce hyperpolarised metabolites, which would cause further losses, the device proposed by Eills *et al*. is not suitable for applications in the life sciences, unless its yield can be improved significantly. This requires quantitative understanding of the kinetics, including the transport phenomena occurring in the chip due to convection and diffusion of reaction species in the flowing liquid as well as the diffusion of $p\;-\;H_{2}$ gas through the membrane. Performance is dictated by the intrinsic kinetics of the reaction and the transport properties of the LoC device.^[18]^ Computational fluid dynamics (CFD) calculations have been used to model and simulate many reactors including micro‐mixers,^[19]^ biosensors[^20^, ^21^] and catalytic reactors.^[22]^ However, to our knowledge, this has not been done in the context of a PHIP reaction. Finite element modelling (FEM) is a powerful method to predict the outcome of a chemical reaction in a microfluidic device without the expense of laboratory time. The model allows to change the geometry of the device, adjust the reaction rates as well as identify rate limiting steps in order to simulate the most favourable conditions for the investigated reaction. A FEM created for a particular geometry can predict the result of any reaction given that the kinetic parameters are known.

In this work, we present a spatially resolved kinetic model of the reaction of propargyl acetate with parahydrogen gas in the presence of a rhodium catalyst in a microfluidic device. The model was developed in two phases. First, a kinetic model of the reaction itself was developed. A set of kinetic equations was derived from the known reaction mechanism.[^23^, ^24^, ^25^] Although kinetic data can be obtained using microfluidic devices, such measurements are much more cumbersome due to limited signal‐to‐noise ratio (SNR) in the microfluidic system with thermal hydrogen. Additionally, the time scale for the formation of propyl acetate is difficult to reach in flow as extremely low flow rates would be required. Therefore, experimental kinetic data was obtained in conventional scale NMR tubes, where thermal hydrogen gas was bubbled through the solution of propargyl acetate and the catalyst, and the evolving concentrations of reaction products were monitored by NMR. In phase two, a coupled convection‐diffusion‐reaction finite element model was developed, using the rate constants obtained from the macroscopic experiments. In the remainder of this paper, the computational model is explained in detail, and the experimental results for the microscopic reaction of propargyl acetate with parahydrogen are discussed. Finally, the resulting FEM calculations are compared to experimental data given by Eills *et al*.^[16]^

## 2 Reaction Mechanism and Kinetic Model

**Figure 1:**
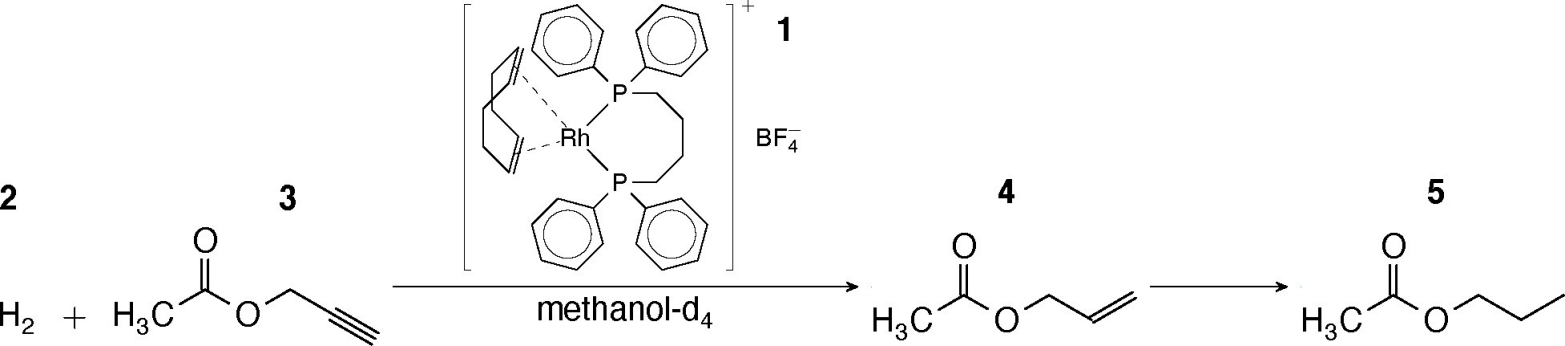
Scheme of the hydrogenation reaction studied. Rh(dpbb)COD **1** catalyses the reaction of hydrogen gas **2** and propargyl acetate **3** to produce allyl acetate **4**. Upon further hydrogenation propyl acetate **5** is formed.

**Figure 2:**
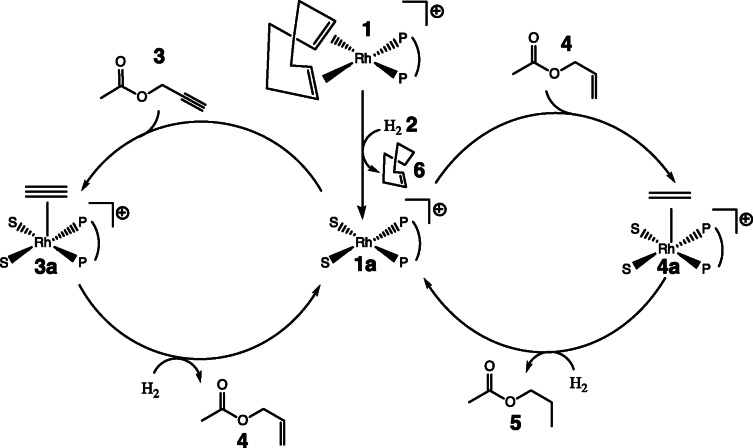
Simplified reaction mechanism for propargyl acatetate hydrogenation displayed in Figure 1. The placement of the unsaturated molecules in species **3** **a** and **4** **a** was drawn schematically for convenience.

The hydrogenation reaction modelled in this work is shown in Figure 1. The first step in the reaction mechanism is the activation of the catalyst precursor. It has been reported that this step involves a coordination of hydrogen to an inactive catalyst complex. This results in a conversion of 1,5‐cyclooctadiene (COD) to cyclooct‐4‐enyl, and is followed by an elimination step. A molecule of cyclooctene (COE) and an active catalyst molecule are produced.^[23]^ The catalytic cycle starts with coordination of the unsaturated precursor molecule.^[24]^ The next step is the oxidative addition of hydrogen into the complex followed by a migratory insertion onto the unsaturated molecule, which results in an alkene. Lastly, in an elimination step, the alkene is released from the complex.^[25]^ This mechanism is too complex to allow for the determination of all rate constants from the experimental data. For this reason, some simplifying assumptions were made for the current model, as shown in Figure 2. The induction period was reduced to a single step, where the complex **1** reacts with a hydrogen molecule **2** to yield an active catalyst complex **1** **a** and cyclooctene **6**. The catalytic cycle starts by propargyl acetate **3** binding to the active catalyst **1** **a** to create complex **3** **a**. Reduction of the catalyst‐bound propargyl acetate to allyl acetate **4** and its elimination from the catalyst was assumed to proceed as a concerted reaction. Since the catalyst is not selective, allyl acetate **4** can re‐enter the cycle and get reduced to propyl acetate **5** in the same manner. This leads to a kinetic model consisting of 5 irreversible reactions, each associated with a forward rate constant:

(1a) $$1+2\overset{k_{1}}{\underset{}{\to }}1a+6$$

(1b) $$1a+3\overset{k_{2}}{\underset{}{\to }}3a$$

(1c) $$3a+2\overset{k_{3}}{\underset{}{\to }}4+1a$$

(1d) $$1a+4\overset{k_{4}}{\underset{}{\to }}4a$$

(1e) $$4a+2\overset{k_{5}}{\underset{}{\to }}5+1a$$

The corresponding rate equations are

(2a) $$\frac{d[1]}{dt}=-k_{1}\left[1\right]\left[2\right],$$

(2b) $$\frac{d[1a]}{dt}=+k_{1}\left[1\right]\left[2\right]-k_{2}\left[1a\right]\left[3\right]+k_{3}\left[3a\right]\left[2\right]-k_{4}\left[1a\right]\left[4\right]+k_{5}\left[4a\right]\left[2\right],$$

(2c) $$\frac{d[2]}{dt}=-k_{1}\left[1\right]\left[2\right]-k_{3}\left[3a\right]\left[2\right]-k_{5}\left[4a\right]\left[2\right],$$

(2d) $$\frac{d[3]}{dt}=-k_{2}\left[1a\right]\left[3\right],$$

(2e) $$\frac{d[3a]}{dt}=+k_{2}\left[1a\right]\left[3\right]-k_{3}\left[3a\right]\left[2\right],$$

(2f) $$\frac{d[4]}{dt}=+k_{3}\left[3a\right]\left[2\right]-k_{4}\left[1a\right]\left[4\right],$$

(2g) $$\frac{d[4a]}{dt}=+k_{4}\left[1a\right]\left[4\right]-k_{5}\left[4a\right]\left[2\right],$$

(2h) $$\frac{d[5]}{dt}=+k_{5}\left[4a\right]\left[2\right],$$

(2i) $$\frac{d[6]}{dt}=+k_{1}\left[1\right]\left[2\right].$$

## 3 Results and Discussion

**Figure 3:**
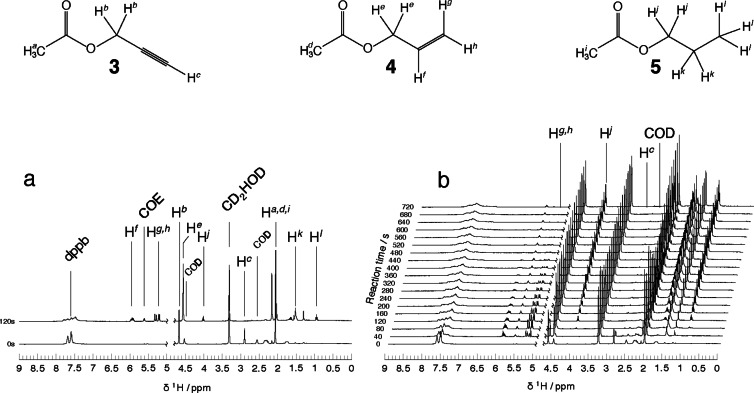
a) Assignment of species present in solution. b) Experimental data obtained by bubbling thermal hydrogen **2** (5 bar) for 10 s through a solution of 20 mM propargyl acetate **3** and 5 mM of catalyst **1**. A transient was acquired every 40 s until 720 s. Methanol peak at 4.78 ppm was suppressed.

Hydrogenation was performed in a valved NMR tube with a threaded capillary to allow for hydrogen gas delivery. The precursor solution contained 20 mM of propargyl acetate **3** and 5 mM of rhodium catalyst **1**. Thermal hydrogen **2** was bubbled at 5 bar for 10 s at $400\;\mathrm{mL}\;\min^{-1}$ then the solution was left to settle for 25 s and a transient was acquired. As a result, a single‐scan proton spectrum was acquired every 40 s. A total of 19 spectra were acquired. Figure 3a shows two spectra at 0 s and 120 s with peak assignments. Figure 3b shows the NMR spectra as the reaction proceeds, with the peaks that display the most significant change labelled. At time=0 s the sample contains propargyl acetate as evidenced by the $H^{c}$ peak at 2.9 ppm. After one bubbling event i.e at 40 s the $H^{c}$ peak has decreased by 60 % and has completely disappeared by 80 s. The conversion of propargyl acetate **3** to allyl acetate **4** is very rapid. The secondary hydrogenation to form propyl acetate **5** is a much slower process. The $H^{j}$ peak firstly appears at 3.9 ppm at 80 s and grows until 640 s, by which point the peaks associated with **4** have completely disappeared.

Catalyst activation can be followed in the region of 7.4 to 7.7 ppm, which corresponds to the aromatic protons on the 1,4‐bis(diphenylphosphino)butane (dppb) ligand. Initially there are two broad peaks, which slowly split to four peaks that are observed at 120 s. The region remains unchanged until 460 s. After 460 s, the four peaks slowly decline and broaden. This may be due to the degradation of the catalyst, possibly by oxidation.

**Figure 4:**
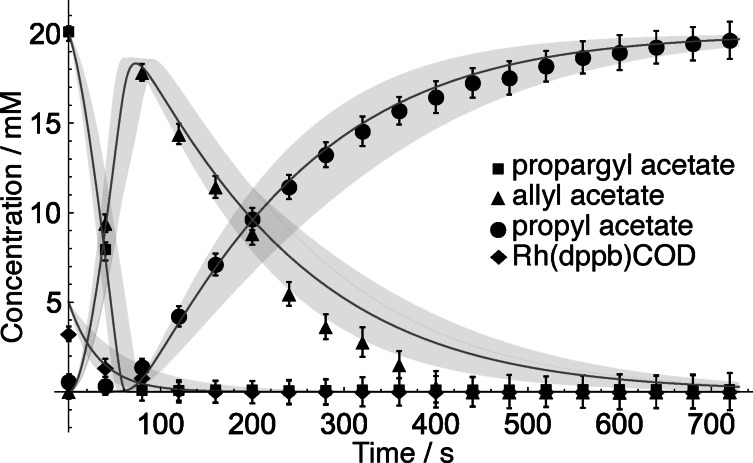
Concentration vs time data of propargyl acetate **3** (square), allyl acetate **4** (triangle), propyl acetate **5** (circle) and the catalyst **1** (diamond). The solid lines represent the best fit to the experimental model, the shadows are the 95 % confidence intervals of the reaction rate constants.

By integrating the $H^{c}$ , $H^{g,h}$ , $H^{j}$ and COD peaks, time‐resolved concentration data was extracted, as shown in Figure 4. Squares indicate propargyl acetate, triangles – allyl acetate, circles propyl acetate and diamonds represent the catalyst region. Error bars were calculated from the small variation in SNR in the spectra.

The conversion from propargyl acetate **3** to allyl acetate **4** and lastly to propyl acetate **5** was simulated by solving differential equations (2a) to (2i), assuming a steady state hydrogen concentration of 20 mM. This assumption was made because hydrogen solubility in methanol at room temperature is 4 mM/bar and experiments were carried out at 5 bar.^[26]^ The starting concentration of **3** was 20 mM and the catalyst **1** was 5 mM; all other initial concentrations were zero. The reaction rate constants were obtained by non‐linear least squares model fitting in Mathematica. Solid lines in Figure 4 represent the best fit of the data; the shaded region corresponds to the boundaries of the 95 % confidence intervals. The resulting reaction rate constants are listed in Table 1 along with their 95 % confidence intervals.

**Table 1:** Reaction equations and rate constants obtained by non‐linear model fit to experimental data with their 95 % confidence intervals.

Reaction	Rate Constant/${\mathrm{mM}}^{-1}\;s^{-1}$	95 % Confidence Interval
**1+2**→**1a**+**6**	$k_{1\;}=\;0.0015$	(0.0008,0.0022)
**1a+3**→**3a**	$k_{2}\;=\;0.5016$	(0.4659,0.5373)
**3a+2**→**1a+4**	$k_{3\;}=\;0.0056$	(0.0035,0.0077)
**1a+4**→**4a**	$k_{4}\;=\;0.0014$	(0.0013,0.0019)
**4a+2**→**1a+5**	$k_{5}\;=\;0.0038$	(0.0013,0.0063)

The catalyst induction period is slow with a rate constant of $k_{1}=\;0.0015\;{\mathrm{mM}}^{-1}\;s^{-1}$ . Once **1 a** reaches sufficient concentration, creation of the catalyst‐substrate complex is comparatively fast, with $k_{2}=\;0.5016\;{\mathrm{mM}}^{-1}\;s^{-1}$ . Elimination of the first hydrogenation product **4** proceeds with a reaction rate constant $k_{3}=\;0.0056\;{\mathrm{mM}}^{-1}\;s^{-1}$ . Coordination of **4** to **1** **a** is a much slower process compared with the production of compound **3** **a** as $k_{4}=\;0.0014\;{\mathrm{mM}}^{-1}\;s^{-1}$ . Lastly, elimination of **5** proceeds with $k_{5}=\;0.0038\;{\mathrm{mM}}^{-1}\;s^{-1}$ . The simulation predicts very well the first hydrogenation event however, there is a minor discrepancy in the prediction of the reduction of allyl acetate to propyl acetate. The experimental data shows a near linear (pseudo‐zeroth order) consumption of **4** from 120 s to 480 s, whereas the simulation predicts a more gradual (pseudo‐first order) decline between 320 s and 600 s. This discrepancy most likely arises due to measurement error.

**Figure 5:**
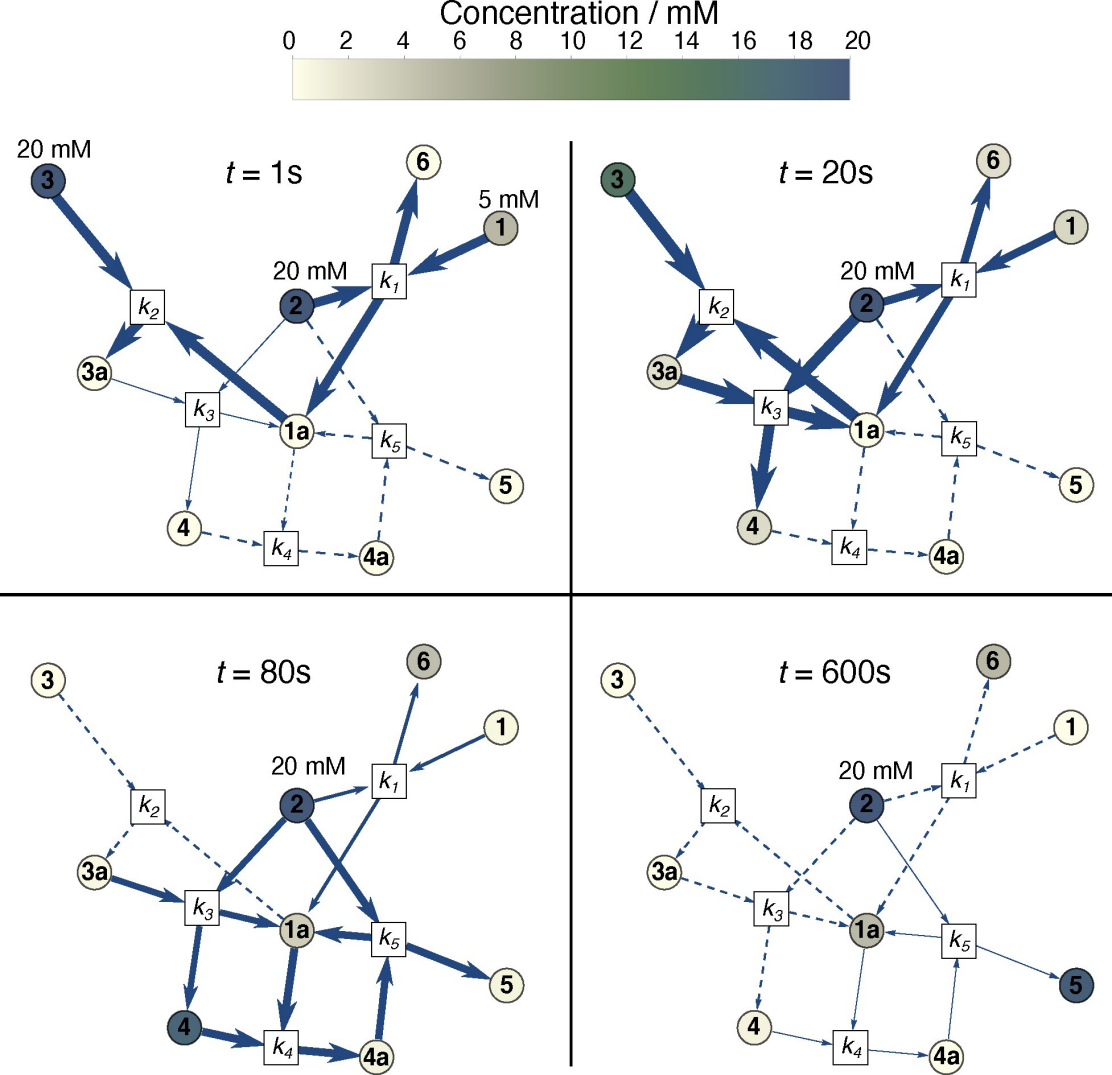
Kinetic model of propargyl acetate hydrogenation reaction. The intensity of the colour from cream to navy indicates the concentration of species. The thickness of the arrow indicates the flux. Dashed arrows correspond to no flux. *t*=1 s the model assumes 20 mM steady state hydrogen **2** concentration, 5 mM of the pre‐catalyst **1** and 20 mM of propargyl acetate **3**. Concentrations of all other species were set to zero. The reaction is dominated by *k*_1_ and *k*_2_. *t*=20 s, early stage of the reaction, *k*_2_ and *k*_3_ are the dominant reaction rate constants. *t*=80, there is very little flux to *k*_1_ and no flux to *k*_2_ while *k*_4_ and *k*_5_ dominate. *t*=600 s is the late stage of the reaction, there is no flux at *k*_1_, *k*_2_ and *k*_3_ while *k*_4_ and *k*_5_ are very slow.

Figure 5 illustrates the reaction network represented by the kinetic model. The intensity of the colour from cream to navy indicates the concentration of species. The arrow thickness corresponds to flux, while a dashed arrow represents no flux. In the early stage of the reaction i.e *t*=1 s, the reaction is dominated by $k_{1}\;\mathrm{and}\;k_{2}$ and there is no flux to $k_{4}\;\mathrm{and}\;k_{5}$ . In the next stage of the reaction *t*=20 s, catalyst activation reaction characterised by *k*_1_ slows down. The reaction is dominated by *k*_2_ and *k*_3_, which correspond to the consumption of propargyl acetate **3** to produce allyl acetate **4**. At *t*=80 s, *k*_4_ and *k*_5_ rates are driving the reaction. In the last stage of the reaction, *t*=600 s there is no flux at *k*_1_, *k*_2_ and *k*_3_ and the reactions driven by *k*_4_ and *k*_5_ are very slow.

**Figure 6:**
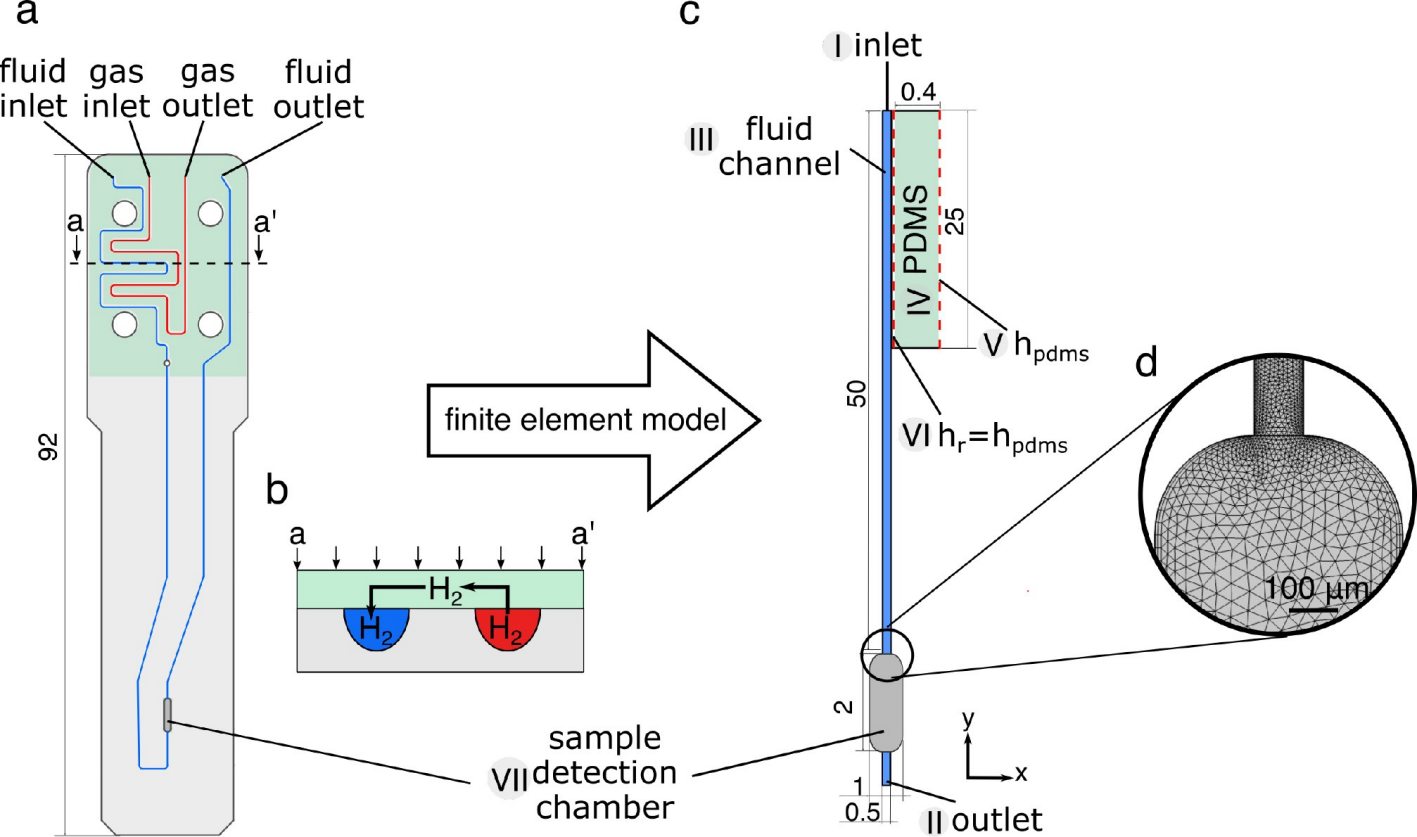
a) The top view of the microfluidic chip. b) Illustration of hydrogen diffusion from the gas channel (red) into the solution channel (blue). PDMS membrane (green) acts as a bridge between the two channels. c) Finite element simulation domain; *h_pdms_* =[**2**]=20 mM is the concentration of hydrogen at outerboundary of the PDMS membrane, *h_r_* is the hydrogen concentration in the fluid channel. d) Mesh at the sample chamber/fluid channel boundary obtained from COMSOL.

The next step involved simulating the mass transport properties of the LoC device. The microfluidic reactor used in Ref. [16] is shown in Figure 6a. The precursor solution containing 20 mM propargyl acetate **3** and 5 mM $\left[\mathrm{Rh}\left(\mathrm{dppb}\right)\mathrm{COD}\right]{\mathrm{BF}}_{4}$ **1** in methanol‐d_4_ is delivered into the fluid channel marked blue via a syringe pump. Hydrogen gas **2** is delivered via a separate channel, marked red. Transport of hydrogen into the liquid channel is facilitated by the use of a semi‐permeable PDMS membrane. The membrane acts as a bridge between the two channels as shown in Figure 6b allowing hydrogen to diffuse into the solution. Reaction products are detected at the 2.5μL sample detection chamber labelled VII. In order to keep computational cost manageable, a 2D finite element representation of the LoC device was constructed rather than a full 3D model. The use of a simple 2D model reduced the computation time to less than 30 minutes and therefore allowed exploration of a wider range of parameters. The simulation domain is shown in Figure 6c and it consists of an inlet I, outlet II, a fluid channel III with a sample chamber VII and a PDMS membrane IV. The flow pattern in the channel was found by solving the Navier‐Stokes equation for incompressible fluids for each flow rate. The resulting velocity distributions were used in a reaction‐diffusion‐convection simulation of the hydrogenation reaction in the channel. The reaction equations and rates were obtained from the space independent model and are listed in Table 1. The gas channel was not modelled explicitly but a constant concentration condition was applied to the outer boundary of the PDMS membrane marked as *h_pmds_* V in Figure 6c. The hydrogen diffusion into the channel was facilitated by coupling the PDMS membrane to the flowing liquid through another concentration condition, marked *h_r_* VI, imposed on the boundary between the membrane and the channel. In order to simulate the volume of the chip, the depth of the domain was fixed to 1.4 mm in the simulation parameters. The 2D model has been designed to ensure that the residence time of the fluid in contact with the PDMS membrane, inside of the transport channel and in the sample chamber agree with the experimental device. Physics‐controlled mesh was automatically generated with the element size set to fine and a sample of the mesh is shown in 6 d.

**Figure 7:**
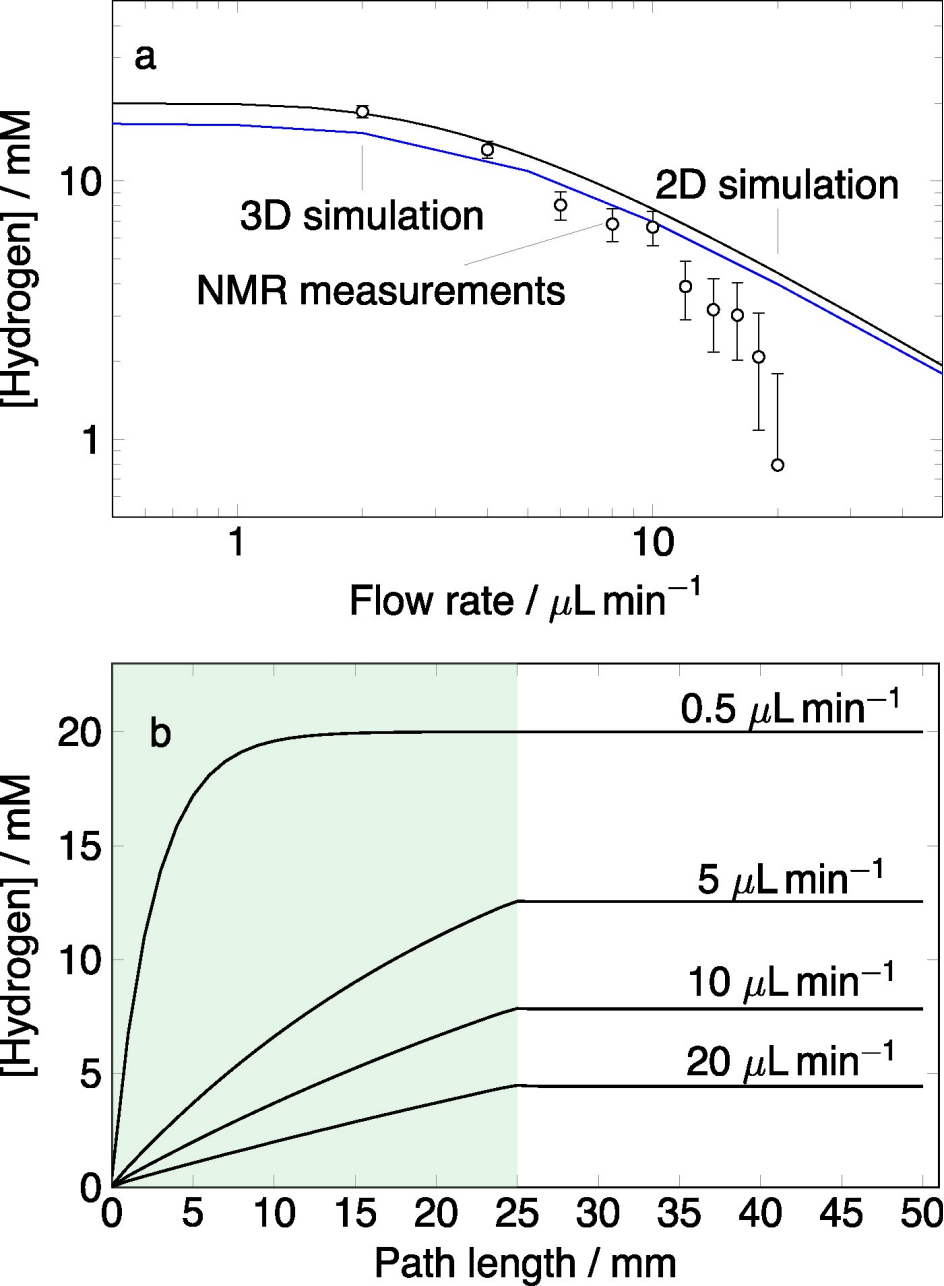
a) Hydrogen concentration in the sample chamber as a function of flow rate. Empty circles correspond to the experimental results and the solid blue line represents the 3D simulation results both obtained from Ref. [16]; the solid black line is the 2D simulation. b) Hydrogen concentration distribution throughout the length of the reaction pathway in the microfluidic chip. The green shading indicates the PDMS membrane.

In a first step, the model was used to predict the uptake of hydrogen into methanol as a function of flow rate, in the absence of catalyst and substrate. This was accomplished by setting all reaction rate constants and the initial concentrations of all species to 0, with the only exception being the hydrogen concentration in the PDMS membrane *h_pmds_* =[**2**]=20 mM. An assumption was made that no hydrogen is lost from the solution after passing the membrane because the residence time of hydrogen in the chip is too short to diffuse through the plastic. Figure 7a shows the concentration of hydrogen in the sample chamber as a function of flow rate. The empty circles correspond to the experimental data reported by Ref. [16]. The solid black line represents the results of the 2D simulation. Experimental data indicates a slow decrease in hydrogen concentration until 10 $\mu L\;\min^{-1}$ . Above 10 $\mu L\;\min^{-1}$ , the concentration steeply declines. The 2D simulation predicts that methanol flowing in the channel is saturated with hydrogen only at low flow rates (below $2\;\mu L\;\min^{-1}$ ). As the flow rate increases, the uptake of hydrogen steadily declines and at high flow rates ($20\;\mu L\;\min^{-1}$ ) only 4.5 mM of hydrogen dissolves in the flowing fluid. Very similar simulation results were presented by Eills *et al*.^[16]^ and are shown in Figure 7a as a solid blue line. In distinction to the current work, 3D calculations were carried out for the full chip geometry. Simulations for the hydrogen flux at the PDMS/liquid interface in the 2D model are in quantitative agreement with the 3D simulations. In both sets of simulations, at flow rates below $10\;\mu L\;\min^{-1}$ the uptake of hydrogen into the fluid is predicted well and the simulated result agrees with the experimental data within the error bars. However, there is an increasing discrepancy at high flow rates. The simulations predict a steady decline of the hydrogen concentration in the sample chamber with an increasing flow rate, whereas the experimental data falls off very rapidly after 10 $\mu L\;\min^{-1}$ . This discrepancy is not yet understood. Eills *et al*.^[16]^ suggested that this could be due to the deformation of the PDMS membrane.

Figure 7b shows the calculated hydrogen concentration distribution in the chip, the green shaded area corresponds to the region where the reaction pathway is in contact with the PDMS membrane. At very low flow rates, $0.5\;\mu L\;\min^{-1}$ hydrogen flux occurs at only at the first few millimeters of the PDMS/liquid boundary before the liquid reaches saturation. As the flow rate increases, the part of the boundary at which flux occurs gradually expands. However, since the amount of liquid per unit time increases as well, this results in a decreasing concentration of hydrogen after contact. At very high flow rates, $20\;\mu L\;\min^{-1}$ this issue becomes prominent with the pathway saturation reaching to only 4.5 mM.

The hydrogenation experiments in a LoC device reported in Ref. [16] were performed using *para*‐enriched hydrogen. Hyperpolarised molecules have a limited life‐time and relax with a time‐constant *T*_1_. The kinetic model proposed in Figure 5 does not account for relaxation of hyperpolarised species as calibration experiments were performed with thermal hydrogen. In order to approximately account for this process, an additional reaction was added:

(3) $$4\overset{k_{6}}{\underset{}{\to }}4rx,\;$$

where *k*_6_ is the nuclear spin‐lattice relaxation rate constant. It has been reported that the ${}^{1}H\;\;T_{1}$ relaxation time of similar compounds is about 7 s, therefore the relaxation rate of *k*_6_ was set to 0.14 $s^{-1}$ .^[27]^ Accounting for the relaxation, results in the following changes to equation 2 f:

(2f‘) $$\frac{d[4]}{dt}=+k_{3}\left[3a\right]\left[2\right]-k_{4}\left[1a\right]\left[4\right]-k_{6}\left[4\right].$$

Moreover, the following equation was added:

(2j) $$\frac{d[4rx]}{dt}=+k_{6}\left[4\right].$$

The remaining equations were unchanged.

**Figure 8:**
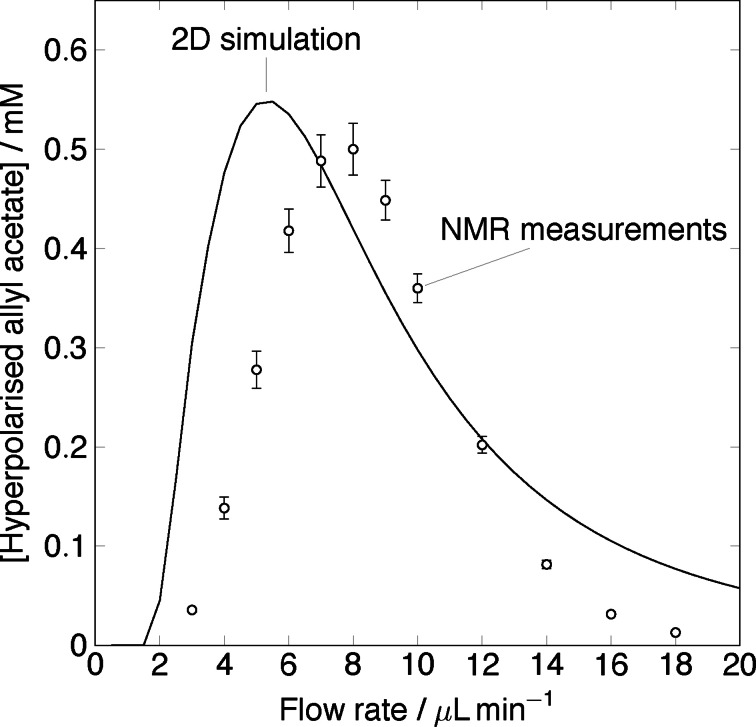
Hyperpolarised allyl acetate concentration in the sample chamber of the chip as a function of flow rate. The empty circles represent the experimental data obtained from Ref. [16] the black solid line is the 2D simulation.

**Figure 9:**
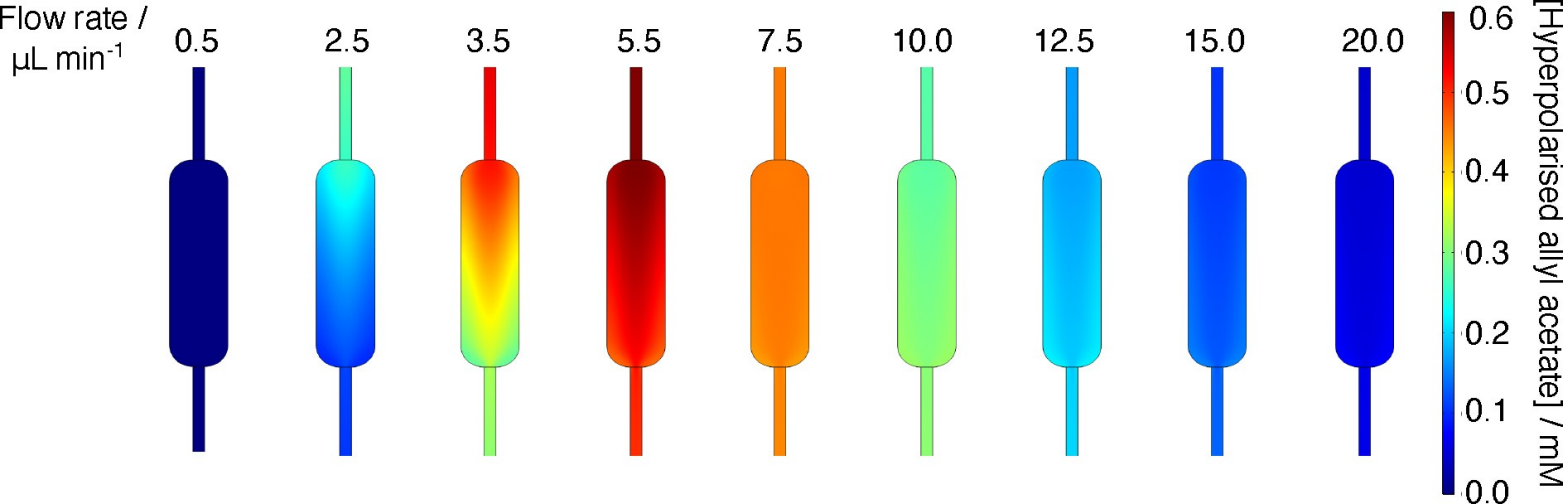
Distribution of hyperpolarised allyl acetate in the sample chamber of the microfluidic chip at varying flow rates.

To perform the finite element simulation, the initial concentrations of propargyl acetate **3** and the catalyst **1** were set to 20 mM and 5 mM respectively (${\left[3\right]}_{0}$ =20 mM, ${\left[1\right]}_{0}$ =5 mM). Hydrogen supply was modelled as a constant concentration condition, *h_pmds_* =[**2**]=20 mM. Initial concentrations of all other species were set to zero. Figure 8 shows the concentration of hyperpolarised allyl acetate at the sample chamber as a function of flow rate. The empty circles represent experimental data obtained from Ref. [16], the black solid line represents the 2D simulation. Experimental data shows that as the flow rate increases from $3\;\mu L\;\min^{-1}$ the concentration of the product increases in the sample chamber until it reaches a maximum at $8\;\mu L\;\min^{-1}$ . Once the maximum is reached, the concentration of hyperpolarised allyl acetate falls rapidly. The simulation predicts a very similar trend, with an initial rise of the product concentration. The maximum is reached at $5.5\;\mu L\;\min^{-1}$ and is followed by a steep decline in the product yield, which tails off at flow rates beyond $12\;\mu L\;\min^{-1}$ . At flow rates below the maximum, the time it takes for the hyperpolarised product to arrive at the sample chamber is greater than its relaxation time. Therefore, at very low flow rates, the product is formed upstream from the sample chamber and on the way, it undergoes relaxation processes. The steep increase in the product from $2\;\mu L\;\min^{-1}$ until the maximum is due to the fact that the product is delivered faster to the sample chamber therefore less of it is lost due to relaxation. At flow rates above the optimum, hydrogen concentration in the reaction channel is less than 10 mM. Low hydrogen concentration results in a significant reduction in the efficiency of the allyl acetate production as two key reactions, namely 1a and 1c, rely on the hydrogen supply. There is a discrepancy in the location of the maximum between experimental data and the simulation. The position of this maximum depends on the chip volume. As discussed previously, the total volume of the chip modelled was calculated based on the chip used by Eills *et al*.^[16]^ Experimental data contains a fabrication error due to imperfect bonding of the chip layers that can result in increased volume of the chip hence different location of the maximum. Figure 9 shows the predicted concentration of hyperpolarised allyl acetate in the sample chamber at different flow rates. At very low flow rates, $0.5\;\mu L\;\min^{-1}$ there is no product being formed in the sample chamber. As the flow rate increases there is an influx into the sample chamber, which can be seen as a colour gradient at flow rates from $2.5\;\mu L\;\min^{-1}$ to $5.5\;\mu L\;\min^{-1}$ . At $5.5\;\mu L\;\min^{-1}$ , a maximum is reached signified by the deep red colour. As the flow rate increases further, the concentration of the product gradually decreases to 0 mM at flow rates of $20\;\mu L\;\min^{-1}$ .

**Figure 10:**
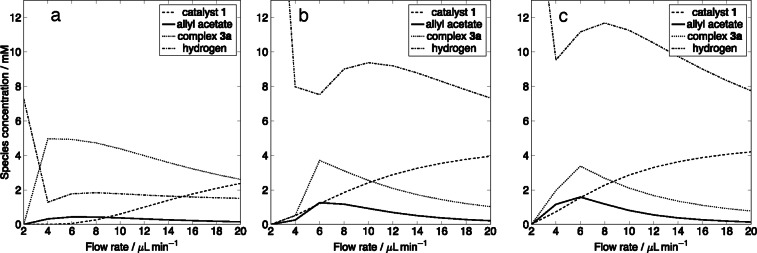
Change in concentration of four key species: protected catalyst **1** (dashed line), hyperpolarised allyl acetate **3** (solid line), complex **3** **a** (dotted line), hydrogen **2** (dash‐dotted line) in the sample chamber as a function of flow rate predicted by the finite element model when a) *k*_1_ was increased tenfold to $k_{1}^{'}$ =0.015 $s^{-1}\;{\mathrm{mM}}^{-1}$ . b) The concentration of hydrogen was doubled to $\left[2\right]$ =40 mM; the reaction rate constants were unchanged. c) The sample detection chamber was moved upstream by 12.5 mm; concentration of hydrogen $\left[2\right]$ =40 mM, reaction rate constants were unchanged.

In order to improve the yield of hyperpolarised allyl acetate in the chip several scenarios were simulated. Figure 10 shows the change in the concentration of four key species: protected catalyst **1** (dashed line), hydrogen **2** (dash‐dotted line), hyperpolarised allyl acetate **3** (solid line) and complex **3** **a** (dotted line), in the sample chamber as a function of flow rate in three scenarios. Since the kinetic model predicts that the slowest step in the formation of allyl acetate is the catalyst deprotection step with *k*_1_=0.0015 $s^{-1}\;{\mathrm{mM}}^{-1}$ , in the simulation, the rate of the reaction was accelerated by increasing *k*_1_ tenfold to $k_{1}^{'}$ =0.015 $s^{-1}\;{\mathrm{mM}}^{-1}$ . Figure 10a shows that all of the available hydrogen is used for the catalyst deprotection step. Therefore, reaction of complex **3** **a** with hydrogen **2** becomes rate limiting. In another simulation, shown in Figure 10 b the concentration of hydrogen was doubled to $\left[2\right]$ =40 mM while reaction rate constants remained unchanged. Doubling the concentration of hydrogen lead to doubling of the hyperpolarised allyl acetate yield however, it remained low at [**3**]=1.2 mM. Hyperpolarised species are subject to relaxation therefore in an attempt to minimise this effect the sample detection chamber was moved upstream by 12.5 mm. The concentration of hydrogen $\left[2\right]$ was kept at 40 mM and the reaction rate constants were unchanged. The result of this simulation is shown in Figure 10c. The yield of hyperpolarised allyl acetate was increased by further 0.5 mM.

The most drastic increase in hyperpolarised allyl acetate production was observed when more hydrogen was supplied to the chip. Therefore devices that maximise uptake of hydrogen needs to be designed.

## 4 Conclusions

In this work a spatially resolved kinetic model of propargyl acetate hydrogenation reaction in a LoC device was developed. In a first step, a kinetic model was developed and was calibrated against independently acquired experimental data. Then, a 2D finite element model of the LoC device was created. The model had no adjustable parameters. In order to test the performance of the model hydrogen flux from from the PDMS membrane was simulated. The simulation successfully predicted the hydrogen uptake at flow rates below $10\;\mu L\;\min^{-1}$ . Above that, the simulation over predicted the amount of hydrogen taken up by the chip. This discrepancy is not yet understood. Following that, the full reaction of allyl acetate hydrogenation was simulated. This was done by utilising reaction rates obtained from macroscopic measurements in a finite element convection‐diffusion‐reaction simulation. The model successfully predicted the yield of allyl acetate in the sample chamber of the device as a function of flow rate. Lastly, the model was used to predict the concentration of hyperpolarised allyl acetate when a few conditions were changed. Overall, in this work we have shown that a simple 2D representation of a microfluidic device can be used to predict the outcome of a hyperpolarised reaction. This a powerful method to test many hypothetical conditions without the expense of laboratory time. The model can be used to test other reactions providing that kinetic parameters are known.

## Experimental Section

All chemicals were purchased from Sigma Aldrich. Experimental data was obtained by dissolving 20 mM (10μL ) propargyl acetate and 5 mM (18 mg) of [1,4‐bis(diphenylphosphino)butane](1,5‐cyclooctadiene)rhodium(I) tetrafluoroborate in 5 mL methanol‐d4. 400μL of the solution was pippeted into a 5 mm pressure valved NMR tube (Norell, UK). The reaction was performed by pressurising the tube with 5 bar of hydrogen (purity 99.995 %) and bubbling the gas at 400 mL $\min^{-1}$ for 10 s inside a 9.1 T Oxford AS400 magnet equipped with Bruker AVANCE Neo console. After bubbling, the sample was left to settle for 25 s and a spectrum was acquired. The nutation frequency for RF pulses was 20.8 kHz for protons. 32 k data points were acquired over 3.3 s for proton 1D spectra. A total of 19 spectra were acquired. The bubbling set up used in this work is described in detail by Dagys *et al*.^[28]^ Concentration profile for species involved in the reaction was obtained by integrating the corresponding region in a ${}^{1}H$ NMR spectrum. Propargyl acetate: 2.85 to 2.95 ppm; allyl acetate 5.00 to 5.50 ppm; propyl acetate 3.97 to 4.06 ppm; catalyst 2.49 to 2.64 ppm. Integrals were calibrated against the concentration of propargyl acetate peak at 2.85 to 2.95 ppm, which was 20 mM.

Rate equations were generated in Mathematica version 12 (Wolfram Research, Inc.) using a home‐built Kinetics Toolbox.^[29]^ Fitting of the experimental data was done using the non‐linear model fit function built into Mathematica.

Finite element simulations were performed using COMSOL Multiphysics version 5.4. The simulation domain was imported from AutoCAD 2019 and is shown in Figure 6b. The domain consisted of a reaction pathway (55 mm length, 0.1 mm width) with a sample chamber (2 mm length, 0.5 mm width) and a PDMS membrane (25 mm length, 0.4 mm width). Entrance thickness was set to $140\times 10^{-5}$ m, which resulted in a chip volume of 8.5 *μL*. All simulation parameters are listed in Table 2. The flow pattern in the reaction pathway was found using the Laminar Flow module and it was done by solving the following Navier‐Stokes equation for laminar flow regime for incompressible fluid:

(4) $$\frac{\partial u}{\partial t}+\nabla \cdot u=0,$$

**Table 2:** Parameters used in COMSOL simulations

Simulation Parameters	
Flow Speed ($\mu L\;\min^{-1}$ )	2...50
Diffusion coefficients ($m^{2}\;s^{-1}$ )	$1\cdot 10^{-9}$
Concentration of Analyte ${\left[1\right]}_{0}$ (mol $m^{-3}$ )	5
Concentration of Analyte ${\left[2\right]}_{0}$ (mol $m^{-3}$ )	20
Concentration of Analyte ${\left[3\right]}_{0}$ (mol $m^{-3}$ )	20
Concentration of Analyte ${\left[1a\right]}_{0},[{2a]}_{0},[3a{{]}_{0},[{4]}_{0},[4a]}_{0},{\left[5\right]}_{0},{\left[6\right]}_{0}$ (mol $m^{-3}$ )	0
Fluid Density *ρ* (kg $m^{-3}$ )	789
Temperature (K)	298.15

where **u** is the flow velocity, *t* is time and ∇· is divergence. To simulate the chemical reactions in the flowing fluid, the velocity distribution from the Laminar Flow module was used as the convection term in the Transport of Dilute Species module using the following equation:

(5) $$\frac{\partial \left[c_{i}\right]}{\partial t}+\nabla \cdot J_{i}+u\cdot \nabla c_{i}=R_{i},$$

Where *c_i_* is the concentration of the species, $J_{i}$ is the mass flux, u is the mass averaged velocity vector and *R_i_* is a reaction rate expression for the species. Reaction rates for 10 species defined in (2a) to (2j) were used with the best fit reaction rate constants obtained from the fitting the space‐independent kinetic model to experimental data (listed in Table 1). The gas channel was not explicitly modelled but a constant hydrogen concentration condition was imposed on the outer boundary of the PDMS membrane shown as $h_{\mathrm{pdms}}=\;\left[2\right]=\;20\;\mathrm{mM}$ . In order to couple the membrane to the reaction pathway, another condition $h_{r}=h_{\mathrm{pdms}}$ was imposed on the boundary between PDMS membrane and the reaction channel.

Physics‐controlled mesh was automatically generated with the element size set to fine. Overall, the mesh consisted of 45210 elements, and the computation involved 312446 independent degrees of freedom. Spatial resolution was approximately 50 μm. PARDISO stationary solver was used.

## Conflict of interest

The authors declare no conflict of interest.
